# Prognostic Value of CD109+ Circulating Endothelial Cells in Recurrent Glioblastomas Treated with Bevacizumab and Irinotecan

**DOI:** 10.1371/journal.pone.0074345

**Published:** 2013-09-12

**Authors:** Lucia Cuppini, Angelica Calleri, Maria Grazia Bruzzone, Elena Prodi, Elena Anghileri, Serena Pellegatta, Patrizia Mancuso, Paola Porrati, Anna Luisa Di Stefano, Mauro Ceroni, Francesco Bertolini, Gaetano Finocchiaro, Marica Eoli

**Affiliations:** 1 Department of Neuro-Oncology Unit, Fondazione IRCCS Istituto Neurologico C, Besta, Milan, Italy; 2 Department of Hematology-Oncology, European Institute of Oncology, Milan, Italy; 3 Department of Neuro-Radiology, Fondazione IRCCS Istituto Neurologico C, Besta, Milan, Italy; 4 Department of Neurology, Fondazione IRCCS Istituto Neurologico Nazionale C. Mondino, Pavia, Italy; University of California-San Francisco, United States of America

## Abstract

**Background:**

Recent data suggest that circulating endothelial and progenitor cells (CECs and CEPs, respectively) may have predictive potential in cancer patients treated with bevacizumab, the antibody recognizing vascular endothelial growth factor (VEGF). Here we report on CECs and CEPs investigated in 68 patients affected by recurrent glioblastoma (rGBM) treated with bevacizumab and irinotecan and two Independent Datasets of rGBM patients respectively treated with bevacizumab alone (n=32, independent dataset A: IDA) and classical antiblastic chemotherapy (n=14, independent dataset B: IDB).

**Methods:**

rGBM patients with KPS ≥50 were treated until progression, as defined by MRI with RANO criteria. CECs expressing CD109, a marker of tumor endothelial cells, as well as other CEC and CEP subtypes, were investigated by six-color flow cytometry.

**Results:**

A baseline count of CD109+ CEC higher than 41.1/ml (1^st^ quartile) was associated with increased progression free survival (PFS; 20 versus 9 weeks, *P*=0.008) and overall survival (OS; 32 versus 23 weeks, *P*=0.03). Longer PFS (25 versus 8 weeks, *P*=0.02) and OS (27 versus 17 weeks, *P*=0.03) were also confirmed in IDA with CD109+ CECs higher than 41.1/ml but not in IDB. Patients treated with bevacizumab with or without irinotecan that were free from MRI progression after two months of treatment had significant decrease of CD109+ CECs: median PFS was 19 weeks; median OS 29 weeks. The presence of two non-contiguous lesions (distant disease) at baseline was an independent predictor of shorter PFS and OS (*P*<0.001).

**Conclusions:**

Data encourage further studies on the predictive potential of CD109+ CECs in GBM patients treated with bevacizumab.

## Introduction

Glioblastomas (GBM) are highly vascularized tumors: several antiangiogenic drugs including bevacizumab (Avastin^®^, F. Hoffmann-La Roche Ltd, Basel, Switzerland), a monoclonal antibody targeting the vascular endothelial growth factor (VEGF), have been investigated for their treatment [1]. The search for predictive markers to select patients who may benefit from treatment is very active [2].

Circulating endothelial and progenitor cells (CECs and CEPs, respectively) are considered with increasing interest as predictive biomarkers [3-5]. CECs, rare in healthy individuals, increase in vascular disorders and tumors due to vascular damage. CEPs are mobilized from the bone marrow to complement local angiogenesis. VEGF is highly expressed in GBM and may mobilize endothelial precursors from the bone marrow [6-8]. In particular, levels of CEPs, defined as CD34+ CD133+ VEGFR2+ cells, were higher in GBM patients than in patients with brain metastases or in controls and correlated with increased density of tumor blood vessels [9]. Variations in the number of pericyte precursors (progenitor perivascular cells, PPCs) could also provide relevant prognostic and possibly predictive information. Progenitor or committed pericytes expressing the platelet-derived growth factor receptor-beta (PDGFR beta) [10] may play a role in shaping the architecture of the vascular niche of an experimental model of glioma [11] and promote endothelial cell survival through induction of autocrine VEGF signaling [12]. Recent data suggest that high pericyte coverage has a negative prognostic impact in clear cell renal cell carcinoma [13].

CECs and CEPs have been considered for their predictive value in patients with colorectal cancer [14-18], breast cancer [19] and non-small cell lung cancer [20]. Although preliminary data are conflicting due to the differences among cancers and the variety of methodologies used to detect cells [3,4], the study of CECs and CEPs also in central nervous system tumors may be relevant for the identification of new insights of pathogenesis. Seaman et al, comparing the vascular trascriptome of normal resting, normal proliferating and tumor endothelial cells, identified CD109 as one of the membrane proteins that are selectively overexpressed on blood vessels during tumor angiogenesis [21]. Thus, we included the analysis of CD109 expressing CEC in our study.

Here, we report on the potential predictive value of CEC, CEP and PPC counts in patients with recurrent GBM treated with bevacizumab and irinotecan, as well as on their clinical and radiological follow-up. The results suggest that CD109+ CECs, in particular, deserve further investigation as potential predictive marker.

## Methods

### Ethics Statement

The study was carried out according to the Italian Decree Law May 8^th^, 2003 allowing treatment of patients with no therapeutic option, with drugs not yet approved by the Italian Regulatory Agency, but with evidence of efficacy in phase II clinical trials. The protocol was approved by the Ethics Committee of the Neurological Institute “Carlo Besta” of Milan and registered in the Institute database (#1/08). All patients gave written informed consent before inclusion in the therapeutic protocol. All clinical investigation were conducted according to the principles expressed in the Declaration of Helsinki.

### Patients

Sixty-eight GBM patients, who previously underwent prior surgery and radiochemotherapy according to the Stupp’s protocol [22], followed by second or third line chemotherapy, were consecutively enrolled from January 14^th^, 2009 and June 1^st^, 2011 (Table 1). Exclusion criteria were as proposed by Vredenburgh et al. [23]. Follow up of patients was carried out until June 2012.

**Table 1:** Patients characteristics at baseline.

**Characteristic**	**No. of pts**		**%**
Gender			
Male	39		57
Female	29		43
Age, yrs			
Median [all pts] (range)		53 (15-76)	
< 40	11		16
40-60	42		62
> 60	15		22
KPS			
Median [all pts] (range)		70 (50-100)	
< 70	19		28
70-80	45		66
90-100	4		6
Histological diagnosis			
De novo GBM	57		84
Secondary GBM	11		16
Time from first diagnosis, mos (range)		13 (4.5-100)	
Disease recurrence			
1^st^/2^nd^/3^rd^	39/26/3		57/39/4
Prior therapy			
1^st^/2^nd^/3^rd^ surgery	68/32/1		100/47/2
Radiotherapy	68		100
Radiosurgery	3		4
1^st^/2^nd^/3^rd^ line chemotherapy	68/29/3		100/43/4
Systemic therapy			
No Dex/Dex<8mg/Dex≥8mg	9/28/31		13/41/46
EIAED therapy	8		12
Tumor volume, cc (range)		26.57 (0.97-173.2)	
Early progression according to RESCUE study [32]	19		28
MRI patterns at baseline			
Local	47		69
Leptomeningeal dissem.	10		15
Distant	10		15
Multifocal	1		2

Abbreviations: cc, cubic centimetres; dissem, dissemination; EIAED, enzyme-inducing anti-epileptic drugs; GBM, glioblastoma multiforme; mos, months; MR, magnetic resonance; pts, patients; yrs, years.

Irinotecan (125 or 340 mg/m^2^, depending on the concomitant use of enzyme-inducing anti-epileptic drugs [EIAED]) and bevacizumab (10 mg/kg) were administered i.v. every 2 weeks until tumor progression, intolerable toxicity, or patient consent withdrawal [23]. Drugs were supplied free by Roche S.p.A. (Monza, Italy) and Hospira S.r.l. (Napoli, Italy). Patients who started treatment with both drugs but showed inadequate bone marrow function (absolute neutrophil count, ANC, ≤2x10^9^/L and platelet count ≤100x10^9^/L), liver dysfunction (aspartate aminotransferase, AST; alanine aminotransferase ALT ≤2.5xULN) or frequent diarrhea continued treatment with bevacizumab alone. Patients were clinically evaluated before treatment and at drug administrations. Toxicities were graded according to CTCAE v 4.0 (Common Terminology Criteria for Adverse Events: http://ctep.cancer.gov/protocolDevelopment/electronic_applications/ctc.htm).

### MRI and response evaluation

Patients underwent conventional contrast-enhanced MRI using a 1.5T MR system (Siemens, Avanto) with an 8 channels head coil at baseline, every 8 weeks or in case of neurological worsening, until tumor progression.

MRI sequences included axial T1 weighted spin-echo (TE/TR=9.1 ms/500 ms, FA=70°, slice thickness=5 mm, no gap, matrix =187x256, FOV=230x187 mm, number of NEX=2), axial turbo spin-echo T2 and proton density weighted (TE/TR=39-79 ms/3500 ms, FA=180°, slice thickness=5 mm, no gap, matrix=256x256, FOV=240x240 mm, NEX=1), coronal FLAIR (TI=2500 ms, TE/TR=121 ms/8000 ms, FA=150°, slice thickness=5 mm, no gap, matrix=149x320, FOV=250x194 mm, NEX=1). After the administration of contrast medium (Gadovist, 0.1 mmol/kg) axial and 3D T1 weighted images (TE/TR=4.24 ms/1160 ms; FA=15°, voxel size 0.90x0.90x0.90, gap 0,45 mm, matrix=192x256 and FOV=230x172.5 mm, NEX=1) were acquired.

MRI evaluation was performed in agreement with RANO criteria [24], by two blinded radiologists. To assess changes of FLAIR hyperintensity a threshold of 25% or more of the maximal cross-sectional area was used. Baseline tumor volumes were determined on 3D post-gadolinium T1 weighted images by manually outlining the enhancing portion of the lesion using MRIcro (http://www.mccauslandcenter.sc.edu/mricro/). To calculate the total enhancing volume of the tumor the number of enhancing voxels was multiplied by the voxel volume.

Disease patterns were characterized as local, distant, and diffuse [25]. Scans showing an increased area of abnormal FLAIR signal in the absence of increased or new enhancement were classified as diffuse recurrence [26].

### Circulating endothelial cells and progenitors analysis

**Figure 1:**
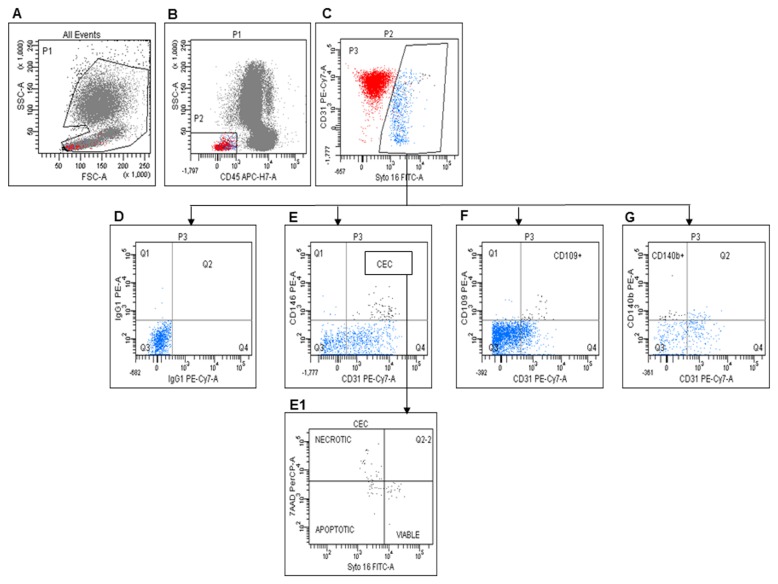
CEC evaluation by flow cytometry. A: Gate used to exclude cell fragments and debris. B: Gate made to identify CD45- cells. C: CD31 expression and Syto16 staining in CD45- cells. D: Negative control for E (CD31+ CD146+, CECs), F (CD31+ CD109+ CECs) and G (CD31-CD140b+, PPCs). E1: Distribution of viable, apoptotic, and necrotic CECs.

Number and viability of CECs and CEPs were measured on fresh samples from 68 patients at baseline and every 8 weeks by six-color flow cytometry. Blood samples were collected in EDTA discarding the first 3 ml of blood to avoid contamination with endothelial cells from venipuncture. The samples were kept at room temperature (22±2 °C) and processed as described in Mancuso et al. [27] within 24 hours after collection. CECs were defined as Syto16(DNA) +CD45-CD31+CD146+ [27]. The combination of Syto16 and 7-AAD was used to discriminate between nucleated viable (Syto16bright/7-AAD-) and apoptotic/necrotic (Syto16dim/7-AAD+) endothelial cells, and to exclude from analysis platelets and endothelial macroparticles. The expression of CD109 in CECs was also investigated. CD109+ CECs were enumerated as Syto16(DNA) +CD45-CD31+CD146+ CD109+ cells and investigated for viability by 7-AAD. Figure 1 shows the CD109+ CEC enumeration procedure.

**Figure 2:**
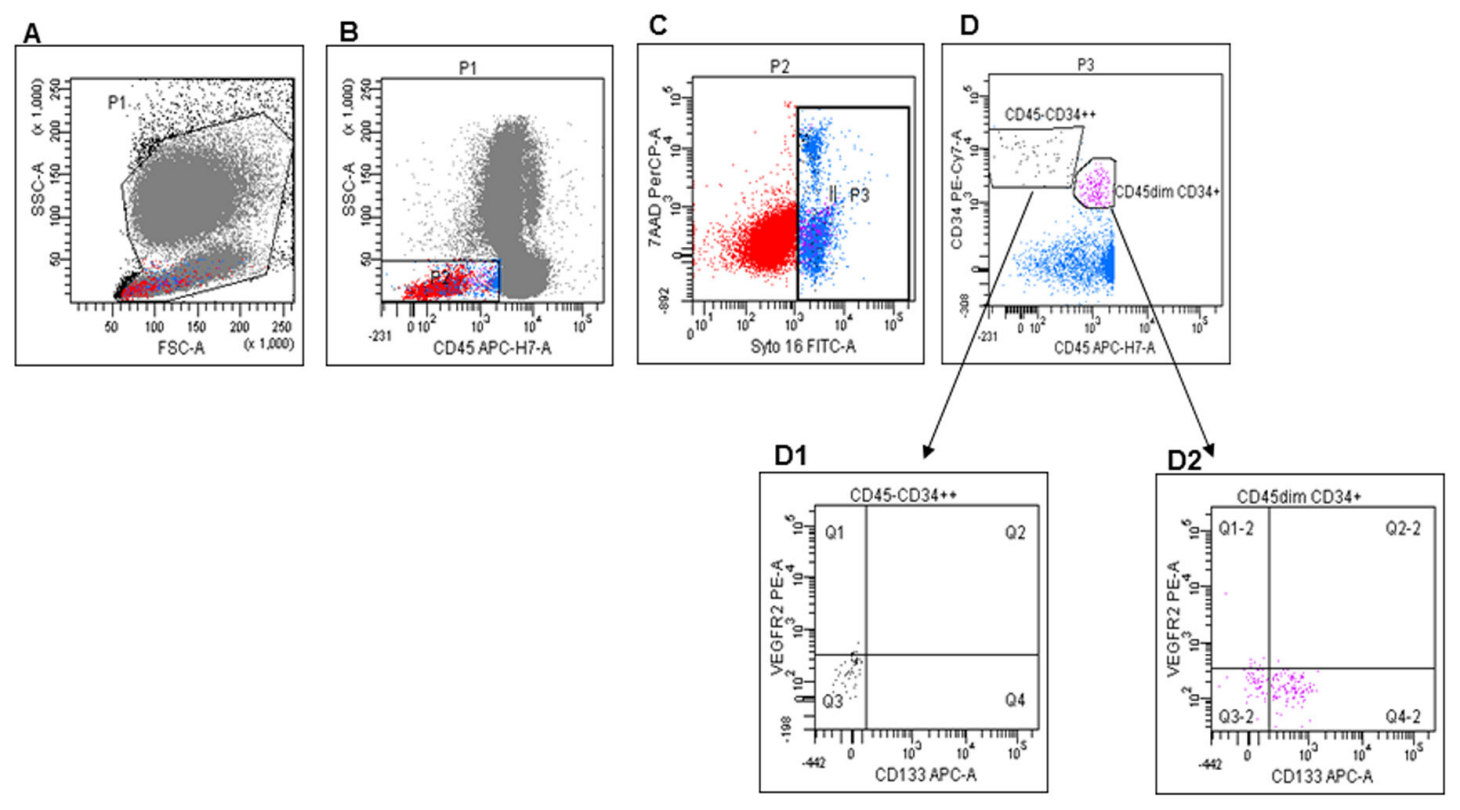
Progenitor cell evaluation by flow cytometry. A: Gate used to exclude cell fragments and debris. B: Gate made to include CD45- and CD45dim cells. C: Gate on Syto16+ 7AAD+ cells. D: Identification of 2 different populations: CD45-CD34+ + and CD133-VEGFR2- (D1), and CD45dimCD34+ and CD133+ cells (D2).

CEPs were evaluated as Syto16(DNA) +CD45-CD34+ [28]. We also investigated the levels of Syto16(DNA) +CD45dimCD34+VEGFR2+, described as VEGFR2+ hematopoietic progenitor cells [29], and of Syto16(DNA) +CD45dimCD34+ and Syto16(DNA) +CD45dimCD34+CD133+ described as hematopoietic committed progenitors (Figure 2) [3,28-30].

As PDGFRbeta (CD140b) + PPCs can differentiate into pericytes and regulate vessel stability and vascular survival in tumors, Syto16(DNA) +CD45-CD31-CD140b+ PPCs were also enumerated [31].

To define reference values, cell populations were investigated also in age- and sex-matched healthy controls. Biological markers of the patient population treated with bevacizumab and irinotecan were compared with two independent datasets. Independent Dataset A (IDA) was formed by 32 age- and sex-matched patients with recurrent GBM and similar clinical characteristics, treated with bevacizumab alone (10 mg/kg i.v., every 2 weeks). Independent Dataset B (IDB) was formed by 14 patients with recurrent GBM, treated with temozolomide (50 mg/mq/die, according to RESCUE study [32]) or fotemustine (75 mg/mq/die on day 1, 8 and 15 followed, after a 35 days interval, by 100 mg/mq on day 1 of a 21 days cycle) constituted a second control group.

### Statistical analysis

Progression Free Survival (PFS) was calculated from treatment onset until disease progression or death/last follow-up, if censored. Overall Survival (OS) was calculated from treatment onset until death/last follow-up, if censored. Kaplan Meier analysis estimated PFS and OS. The log rank test assessed differences in progression or survival in patients with different clinical, radiological or biological parameters. These parameters were set at the 25°, 50°, 75°, 90° percentile and separately evaluated in all patients.

Correlations between biological markers and clinical parameters or treatment response were assessed using the Mann-Whitney exact U test. The Wilcoxon rank sum test evaluated differences among biological markers levels at baseline, week 8 or progression. All *P* values were two-sided.

A multivariate analysis and a Cox proportional hazard regression model analysis were performed on variables showing statistically significant differences at univariate analysis to investigate their independent prognostic role. In particular, CD109+ CECs was used as a dichotomic parameter. All statistical analyses were performed using STATA software 10.0.

## Results

### Treatment

Clinical characteristics of the patients are described in Table 1. None of them was previously treated with bevacizumab or other anti-angiogenic drugs. Fifteen (22%) experienced progression during the first six cycles of adjuvant TMZ therapy according to the Stupp regimen [22]. Two patients progressed <12 weeks after radiation therapy and performed a second MRI confirming progression after 6 weeks; no patient had pseudo-progression.

Fifty-five patients were treated with both bevacizumab and irinotecan until progression; 13 patients interrupted irinotecan before progression due to low tolerance.

IDA and IDB patient characteristics are outlined in Table S1A and B.

### Toxicity

Six patients stopped treatment before disease progression due to: intra-tumoral bleeding (n=2), sub-galeal infection after surgery (n=2), thrombosis of the cerebral sinus (n=1) and consent withdrawal (n=1). Dates of their disease progression and death were included in the statistical analysis.

Five patients died before disease progression due to: pancreatic neoplasia (n=1); ischemic heart failure (n=1); cerebral sinus thrombosis (n=1); unknown reasons (n=2). A detailed list of adverse events with relative grades is provided in Table S2.

### Response rate and patterns of radiological progression

Using RANO criteria [24] we found that 14 patients had a partial response, 40 stable disease and 14 disease progression at week 8; no other partial or complete response was observed later. Patients with partial response at week 8 had longer PFS and OS than the others (39 versus 15 weeks, *P*=0.002; 58 versus 26 weeks, *P*=0.002). Figures S1 and S2, respectively, show one example of tumor progression and one example of partial response 8 weeks after treatment onset.

The analysis of progression patterns excluded patients who died or interrupted treatment before progression and patients with incomplete neuroimaging. Eleven patients converted to a diffuse pattern of disease (9 patients starting from local pattern and 2 from leptomeningeal dissemination), one patient with local disease converted to leptomeningeal dissemination and 36 patients did not show changes when compared to baseline.

### Survival

Median follow up was 28 weeks (5-112 weeks). Median OS was 29 weeks (5-112 weeks); OS at 6 months (OS-6) and 12 months (OS-12) were 58% (95% CI 46-70%) and 23% (95% CI 13-33%), respectively. Five patients were still progression-free at the end of the follow-up. Median PFS was 19 weeks (5-112 weeks). PFS-6 and PFS-12 were 36% (95% CI 24-47%) and 13% (95% CI 5-21%), respectively.

Clinical features potentially affecting PFS and OS are outlined in Table 2. Although tumor volumes in patients with leptomeningeal dissemination or distant tumors were not significantly larger than in other subjects (median volumes 40.1 cc and 44.0 cc, respectively), both subgroups had shorter PFS and OS. Patients assuming <8 mg dexamethasone at baseline had longer PFS and OS than the others.

**Table 2:** Univariate analysis of the most relevant clinical and biological parameters.

	**Median PFS**		***P* value**	**Median OS**		***P* value**
	Wks			wks		
KPS ≤70 versus >70	16	19	n. s.	29	30	n. s.
Age ≤40 yrs versus >40	19	17	n. s.	29	27	n. s.
Age ≤60 yrs versus >60	20	18	n. s.	27	29	n. s.
De novo versus secondary tumor	18	18	n. s.	27	29	n. s.
Tumor volume ≤10.9 mm^3^ versus >10.9 mm^3^	29	15	n. s.	37	24	n. s.
Dex <8 mg versus dex ≥8 mg	29	10	0.0001	39	24	0.002
EIAED use versus EIAED free	19	17	n. s.	29	27	n. s.
Distant disease versus no distant disease	9	26	0.0001	19	38	0.0001
Leptomeningeal diss. versus no leptomeningeal diss.	10	20	0.01	19	31	0.01
CD109+CEC ≤41.1/ml versus >41.1/ml	9	20	0.008	23	32	0.03

Abbreviations: dex, dexamethasone; diss, dissemination; EIAED, enzyme-inducing anti-epileptic drugs; wks, weeks; yrs, years.

### Circulating Endothelial or Progenitor cells

**Figure 3:**
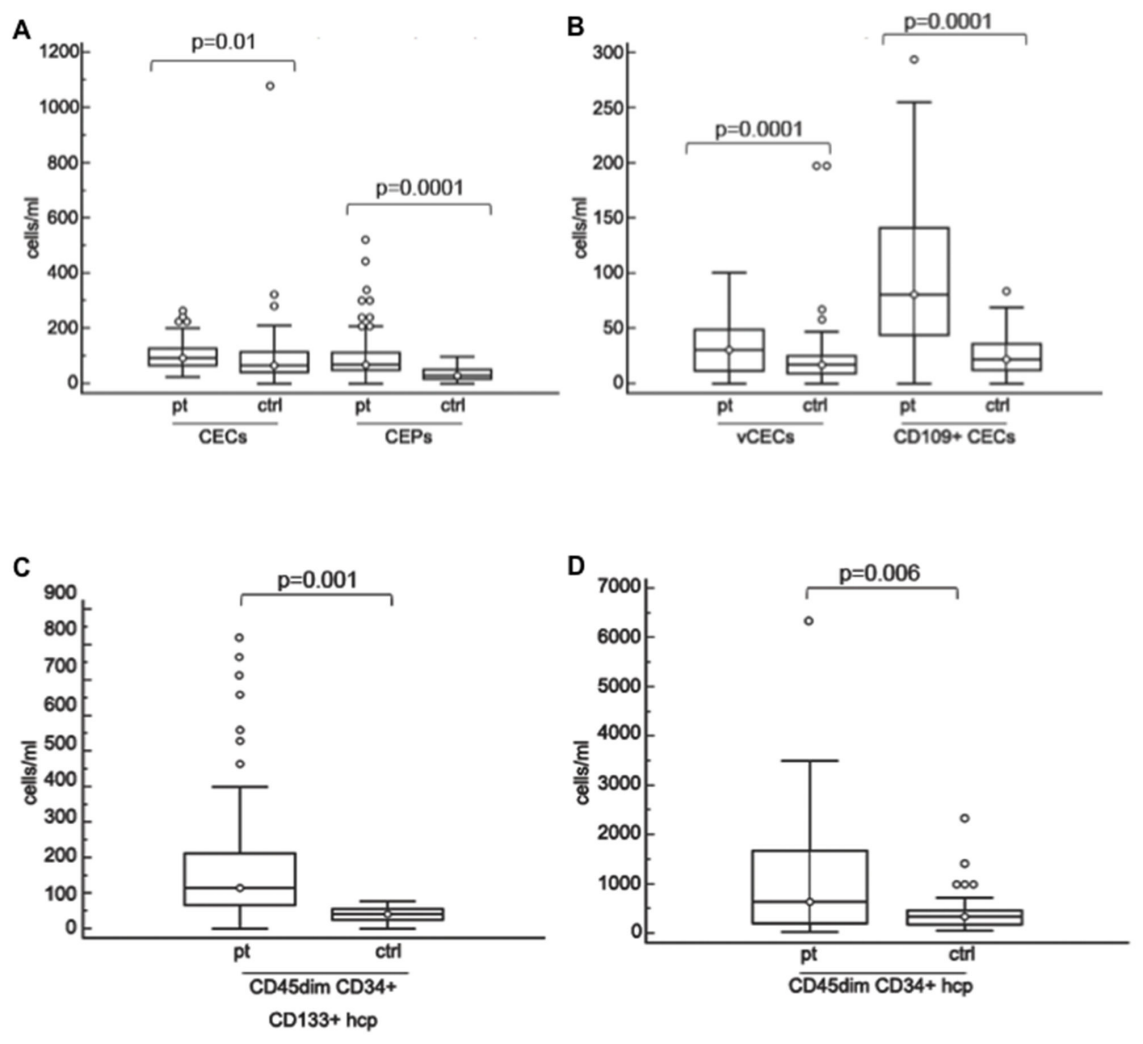
Baseline levels of cell subpopulations in patients treated with bevacizumab and irinotecan and healthy controls. A: Baseline levels of CECs and CEPs in patients and healthy controls. B: Baseline levels of viable CECs and CD109+ CECs in patients and healthy controls. C: Baseline levels of CD45dimCD34+CD133+ hematopoietic progenitors in patients and healthy controls. D: Baseline levels of CD45dimCD34+ hematopoietic committed progenitors in patients and healthy controls. Boxes: the interquartile range; lines: location of first quartile, median, and third quartile; ○, outliers beyond the standard span. All P values were calculated by the Mann-Witney test. Abbreviations: CECs, circulating endothelial cells; vCECs, viable CECs; CEPs, circulating endothelial progenitors; ctrls, healthy controls; hcp, hematopoietic committed progenitors.

Significantly higher levels of CECs (*P*=0.01), CEPs (*P*=0.0001), viable CECs (*P*=0.0001), CD109+ CECs (*P*=0.0001), CD45dimCD34+CD133+ (*P*=0.001) and CD45dimCD34+ (*P*=0.006) hematopoietic committed progenitors were found at baseline in patients treated with bevacizumab and irinotecan, compared to healthy controls, even if *P* values were adjusted for multiple comparisons (Figure 3). Lower levels of CD140b+ PPCs were detected in patients when compared to healthy controls (*P*=0.03, data not shown). No correlation was observed between counts of each cell subpopulation and clinical or radiological parameters (data not shown).

Baseline levels of all cell subpopulations were not significantly different among patients treated with bevacizumab and irinotecan, bevacizumab alone (IDA) or chemotherapy alone (IDB) (data not shown).

**Figure 4:**
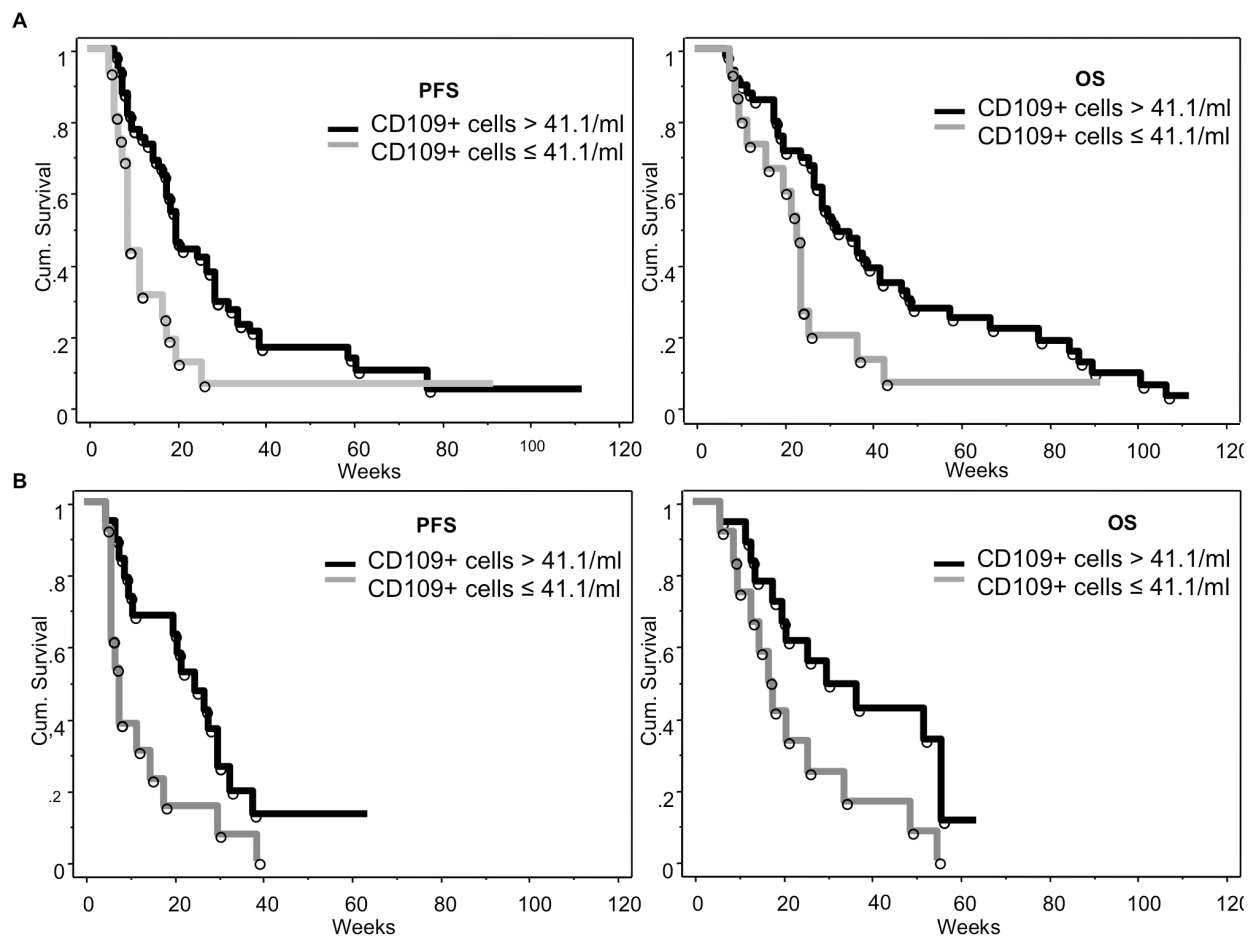
Correlation between baseline CD109+ CECs and PFS/OS in patients treated with bevacizumab+irinotecan or bevacizumab alone. Patients treated with bevacizumab and irinotecan and showing baseline CD109+ CEC count > 41.1/ml (1^st^ quartile) had increased PFS and OS (panels A); PFS was significantly increased also in patients belonging to IDA and baseline CD109+ CEC count over the 1^st^ quartile (panels B).

Patients treated with bevacizumab and irinotecan showing baseline counts of CD109+ CECs higher than 41.1/ml (1^st^ quartile) had increased PFS and OS (Table 2 and Figure 4A); these patients did not show differences of clinical and radiological parameters when compared to the others (Table S3). PFS and OS were significantly increased also in patients treated with bevacizumab alone and baseline counts of CD109+ CEC higher than 41.1/ml (Figure 4B), but not in patients treated with antiblastic chemotherapy (Figure S3).

**Figure 5:**
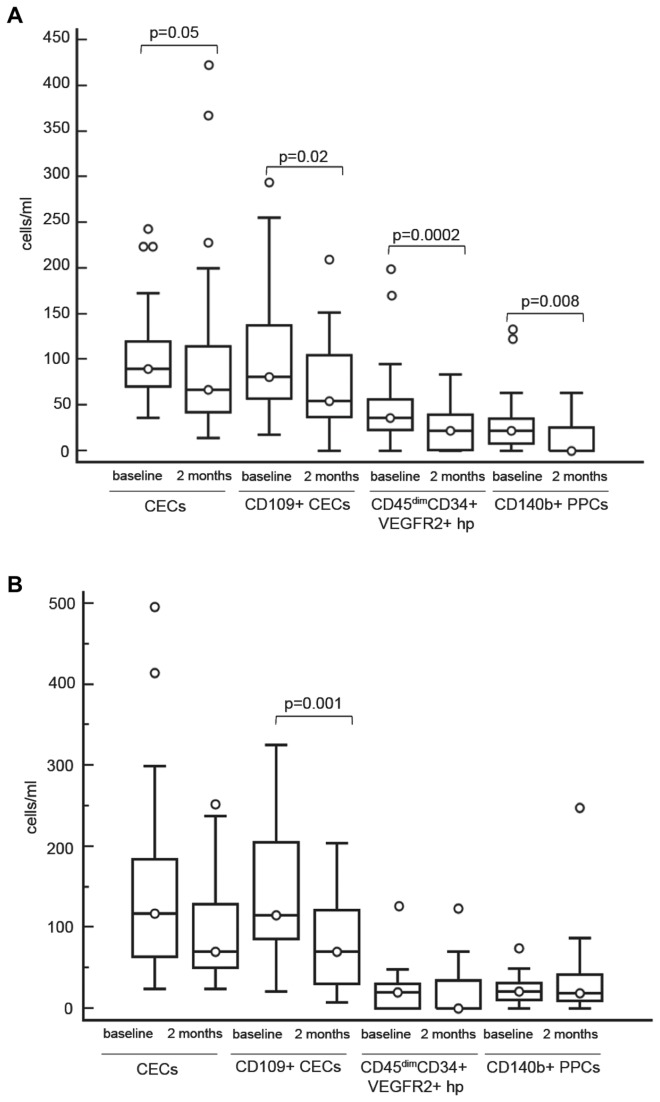
CECs, CD109+ CECs, CD45dimCD34+VEGFR2+ hp, CD140b+ PPCs before therapy and at 2 months. A: Counts of CECs, CD109+ CECs, CD45dimCD34+VEGFR2+ hp, CD140b+ PPCs before therapy and 2 months after treatment onset in patients responding to treatment with bevacizumab and irinotecan. B: Counts of CECs, CD109+ CECs, CD45dimCD34+VEGFR2+ hp, CD140b+ PPCs before therapy and 2 months after treatment onset in patients responding to treatment with bevacizumab alone (IDA). Boxes: the interquartile range; lines: location of first quartile, median, and third quartile. ○, outliers beyond the standard span. All P values were calculated by Wilcoxon test. Abbreviations: CECs, circulating endothelial cells; hp, hematopoietic progenitor cells; PPCs, progenitor perivascular cells; VEGFR, vascular endothelial growth factor receptor.

In patients treated with bevacizumab and irinotecan, different changes in cell counts were detected in patients who progressed at 2 months, here defined as non-responders (n=14), compared to others (responders, n=42): counts of CECs, viable CECs, CD109+ CECs, CD45dimCD34+VEGFR2+ hematopoietic progenitor cells and CD140b+ PPCs decreased significantly after treatment in responders only (Figure 5A). A reduction of CD109+ CECs was also observed in 17 patients responding to treatment with bevacizumab alone (IDA, Figure 5B), whereas in patients treated with chemotherapy (IDB) no reduction of CD109+ CECs (78.3±38 versus 85.7±63, *P*=n.s.) or other cell subpopulation variations were detected.

### Multivariate analysis of patients treated with bevacizumab and irinotecan

The multivariate analysis of the same biological, clinical and radiological parameters influencing PFS or OS at univariate analysis, showed that distant disease at baseline was associated to shorter PFS (*P*=0.001, RR 3.5, 95% CI 1.6-7.4) and OS (*P*=0.003, RR 3.3, 95% CI 1.5-7.2). On the contrary CD109+ CEC counts >41.1/ml at baseline affected positively PFS (*P*=0.03, RR 0.39, 95% CI 0.2-0.7) and OS (*P*=0.04, RR 0.5, 95% CI 0.3-1). PFS only was also decreased in association to leptomeningeal dissemination (*P*=0.04, RR 2.0, 95% CI 1-4.2) and increased when dosage of dexamethasone was <8 mg per day (*P*=0.01, RR 0.4, 95% CI 0.2-0.8). All these variables were independent.

When PFS and OS of patients with baseline counts of CD109+ CECs higher or lower than 41.1/ml were adjusted for dexamethasone dosage (≥8 or <8 mg/die), the difference still remained significant (*P*=0.009 and *P*=0.04, respectively).

## Discussion

Our data show that CECs and CEPs at baseline are higher in GBM patients than in healthy controls, in agreement with previous results on other cancers [4,33]. Treatment with bevacizumab and irinotecan caused a general decrease of CECs and CEPs: however, such decrease was only significant in patients that did not show progression after two months. Our report suggests for the first time a potential link between CD109 expression in CECs and anti-VEGF treatment: baseline CD109+ CEC count >41.1/ml identified a subgroup of patients with longer PFS and OS and were significantly higher in long-term responders than in other patients.

CD109, a glycosylphosphatidylinositol-anchored cell surface glycoprotein [34], is highly expressed in several solid tumors [35] and is one of 12 endothelial markers more expressed in tumor than normal endothelial cells [21]. Treatment with bevacizumab and irinotecan also decreased viable PPC counts, maybe due to the block of PPCs recruitment from tissue *reservoirs* or to PPCs inclusion into blood vessels to repair the damage induced by chemotherapy [27,36,37].

The observation that patients undergoing progression did not display changes of CECs, CD140b+ PPCs and CD45dimCD34+VEGFR2+ hematopoietic progenitor cells suggest that other biological programs, possibly favoring migration and invasion, are involved in progression, as proposed by preclinical studies [38,39].

Median and 6-month PFS and OS were slightly lower than previously reported [40,41], a likely consequence of the enrolment of patients with poor prognostic factors (e.g. KPS > 50; Table 1). OS of our patients is similar to that referred by Desjardins et al. who treated seriously impaired recurrent GBM patients with bevacizumab and metronomic TMZ [42]. PFS and OS in patients treated with bevacizumab and irinotecan were higher than in patients treated with bevacizumab alone, but the difference was not significant, as also found by Friedman et al. [43].

RANO radiological criteria were used for assessment of disease response to treatment [24], as they are better suited to study the effects of anti-angiogenic factors. The observation that distant disease at baseline is associated to a worst prognosis is of interest. Distant disease did not imply a larger tumor volume: thus, this result may allude to an increased resistance to bevacizumab due to the activation of migration/invasion programs that appear somehow alternative to progression programs based on increased angiogenesis [44]. The pattern of recurrence observed in our patients is partially different from those previously reported; at baseline local disease was less frequent than in other reports (68% versus 80% or 72% reported by Chamberlain and Pope, respectively [25,45]) and leptomeningeal dissemination was present in 14% of our patients (6.2% in Chamberlain et al. [25]). At recurrence 24% of our patients converted to a diffuse pattern (30.3% in Pérez-Larraya [46]); moreover, half of patients with leptomeningeal dissemination at baseline developed a diffuse pattern (6% of all patients).

CD109 is a monomeric cell surface glycoprotein of 170 kD that is expressed on endothelial cells, activated T-lymphocytes and platelets and a subpopulation of bone marrow CD34+ cells [47]. Its expression is also high in different cancers and cancer lines, including glioblastoma [35]. CD109 is a TGF-beta co-receptor that regulates TGF-β receptor endocytosis and degradation, thus inhibiting TGF-β signaling [48]. Both VEGF and TGF-beta are involved in the regulation of endothelial cell stability [49]: whether this relationship is relevant to the results we obtained remains to be investigated.

This preliminary study was not powered to adhere to all the criteria for marker identification included in the REMARK checklist [50]. However we believe that the data encourage a larger study on the predictive potential of CD109+ CEC in GBM and possibly other cancer patients treated with bevacizumab. It is conceivable that the combination other potential markers of interest, like baseline plasma levels of VEGF-A, possibly associated with dynamic contrast-enhanced magnetic resonance imaging (DCE-MRI) [51] may help profiling with increased precision patients that may benefit from bevacizumab.

## Supporting Information

Figure S1 **MRI of one patient with tumor progression (A, B, C before treatment. D, E, F at 2 months).** From left to right: axial T1-weighted image (T1WI) with contrast injection, axial T2WI and coronal Flair image. A: recurrent GBM with irregular and marked enhancement and cystic-necrotic areas and invasion of genu of the corpus callosum. Small areas of enhancement are visible in the basal ganglia region bilaterally. B and C: the corresponding T2 and Flair showing heterogeneous hypersignal. The surgical cavity is visible in C. D: marked reduction of the enhancement in the left frontal region and corpus callosum, almost complete disappearance of enhancement in basal ganglia region and lowering of the mass effect. E and F: T2 hypersignal is increased and infiltration of contralateral frontal regions and a slight left hyperintensity in the temporal lobe are also visible.
(TIF)

Figure S2 **MRI of one patient responding to treatment (A, B, C before treatment. D, E, F at 2 months).** From left to right: axial T1- weighted with contrast injection, axial T2-weighted, sagittal Flair. A: left frontal GBM characterized by strong and irregular enhancement. B: the tumor shows heterogeneous signal on T2-wi. C: On Flair images a large T2 hyperintensity surrounding the tumor is visible. The mass effect is demonstrated by narrowing of the cortical sulci and left lateral ventricle compression. D: after two months of therapy the enhancing tumor is dramatically reduced, the left lateral ventricle is slightly enlarged. E, F: only a small hyperintensity is seen on T2-wi and Flair images. No mass effect is visible and the sulci are clearly recognizable.
(TIF)

Figure S3 **Baseline CD109+ CECs and PFS/OS in patients treated with classical antiblastic chemotherapy (IDB).** Baseline CD109+ CEC count > 41.1/ml (1^st^ quartile) were not associated with increased PFS or OS in IDB patients.
(TIF)

Table S1 **IDA patient characteristics.**
(DOC)

Table S2 **Adverse events.**
(DOCX)

Table S3 **Characteristics of patients with CD109+ CEC > 41.1/ml or ≤ 41.1/ml at baseline.**
(DOCX)

## References

[1] Iwamoto FM, Fine HA (2010) Bevacizumab for malignant gliomas. Arch Neurol 67: 285-288. doi:10.1001/archneurol.2010.11. PubMed: 20212225. 20212225 10.1001/archneurol.2010.11 PMC7243880

[2] Jubb AM, Harris AL (2010) Biomarkers to predict the clinical efficacy of bevacizumab in cancer. Lancet Oncol 11: 1172-1183. doi:10.1016/S1470-2045(10)70232-1. PubMed: 21126687. 21126687 10.1016/S1470-2045(10)70232-1

[3] Bertolini F, Shaked Y, Mancuso P, Kerbel RS (2006) The multifaceted circulating endothelial cell in cancer: Towards marker and target identification. Nat Rev Cancer 6: 835-845. doi:10.1038/nrc1971. PubMed: 17036040. 17036040 10.1038/nrc1971

[4] Bertolini F, Mancuso P, Shaked Y (2011) Circulating endothelial cells as biomarkers for patients receiving bevacizumab. Lancet Oncol 12: 217-218. doi:10.1016/S1470-2045(11)70050-X. PubMed: 21376289. 21376289 10.1016/S1470-2045(11)70051-1

[5] Calleri A, Bono A, Bagnardi V, Quarna J, Mancuso P et al. (2009) Predictive potential of angiogenic growth factors and circulating endothelial cells in breast cancer patients receiving metronomic chemotherapy plus bevacizumab. Clin Cancer Res 15: 7652-7657. doi:10.1158/1078-0432.CCR-09-1493. PubMed: 19996223. 19996223 10.1158/1078-0432.CCR-09-1493

[6] Plate KH, Breier G, Weich HA, Risau W (1992) Vascular endothelial growth factor is a potential tumour angiogenesis factor in human gliomas in vivo. Nature 359: 845-848. doi:10.1038/359845a0. PubMed: 1279432. 1279432 10.1038/359845a0

[7] Li B, Sharpe EE, Maupin AB, Teleron AA, Pyle AL et al. (2006) VEGF and PlGF promote adult vasculogenesis by enhancing EPC recruitment and vessel formation at the site of tumor neovascularization. FASEB J 20: 1495-1497. doi:10.1096/fj.05-5137fje. PubMed: 16754748. 16754748 10.1096/fj.05-5137fje

[8] Asahara T, Takahashi T, Masuda H, Kalka C, Chen D et al. (1999) VEGF contributes to postnatal neovascularization by mobilizing bone marrow-derived endothelial progenitor cells. EMBO J 18: 3964-3972. doi:10.1093/emboj/18.14.3964. PubMed: 10406801. 10406801 10.1093/emboj/18.14.3964 PMC1171472

[9] Rafat N, Beck GC, Schulte J, Tuettenberg J, Vajkoczy P (2010) Circulating endothelial progenitor cells in malignant gliomas. J Neurosurg 112: 43-49. doi:10.3171/2009.5.JNS081074. PubMed: 19522573. 19522573 10.3171/2009.5.JNS081074

[10] Song S, Ewald AJ, Stallcup W, Werb Z, Bergers G (2005) PDGFRbeta+ perivascular progenitor cells in tumours regulate pericyte differentiation and vascular survival. Nat Cell Biol 7: 870-879. 16113679 10.1038/ncb1288 PMC2771163

[11] Bababeygy SR, Cheshier SH, Hou LC, Higgins DM, Weissman IL et al. (2008) Hematopoietic stem cell-derived pericytic cells in brain tumor angio-architecture. Stem Cells Dev 17: 11-18. doi:10.1089/scd.2007.0117. PubMed: 18240955. 18240955 10.1089/scd.2007.0117

[12] Franco M, Roswall P, Cortez E, Hanahan D, Pietras K (2011) Pericytes promote endothelial cell survival through induction of autocrine VEGF-A signaling and bcl-w expression. Blood 118: 2906-2917. doi:10.1182/blood-2011-01-331694. PubMed: 21778339. 21778339 10.1182/blood-2011-01-331694 PMC3172806

[13] Cao Y, Zhang ZL, Zhou M, Elson P, Rini B et al. (2012) Pericyte coverage of differentiated vessels inside tumor vasculature is an independent unfavorable prognostic factor for patients with clear cell renal cell carcinoma. Cancer, 119: 313–24. PubMed: 22811049. 22811049 10.1002/cncr.27746

[14] Ronzoni M, Manzoni M, Mariucci S, Loupakis F, Brugnatelli S et al. (2010) Circulating endothelial cells and endothelial progenitors as predictive markers of clinical response to bevacizumab-based first-line treatment in advanced colorectal cancer patients. Ann Oncol 21: 2382-2389. doi:10.1093/annonc/mdq261. PubMed: 20497963. 20497963 10.1093/annonc/mdq261

[15] Simkens LH, Tol J, Terstappen LW, Teerenstra S, Punt CJ et al. (2010) The predictive and prognostic value of circulating endothelial cells in advanced colorectal cancer patients receiving first-line chemotherapy and bevacizumab. Ann Oncol 21: 2447-2448. doi:10.1093/annonc/mdq640. PubMed: 21030382. 21030382 10.1093/annonc/mdq640

[16] Murakami H, Ogata Y, Akagi Y, Ishibashi N, Shirouzu K (2011) Circulating endothelial progenitor cells in metronomic chemotherapy using irinotecan and/or bevacizumab for colon carcinoma: Study of their clinical significance. Exp Ther Med 2: 595-600. PubMed: 22977546. 22977546 10.3892/etm.2011.253 PMC3440687

[17] Matsusaka S, Suenaga M, Mishima Y, Takagi K, Terui Y et al. (2011) Circulating endothelial cells predict for response to bevacizumab-based chemotherapy in metastatic colorectal cancer. Cancer Chemother Pharmacol 68: 763-768. doi:10.1007/s00280-010-1543-2. PubMed: 21170650. 21170650 10.1007/s00280-010-1543-2

[18] Malka D, Boige V, Jacques N, Vimond N, Adenis A et al. (2012) Clinical value of circulating endothelial cell levels in metastatic colorectal cancer patients treated with first-line chemotherapy and bevacizumab. Ann Oncol 23: 919-927. doi:10.1093/annonc/mdr365. PubMed: 21825101. 21825101 10.1093/annonc/mdr365

[19] Bidard FC, Mathiot C, Degeorges A, Etienne-Grimaldi MC, Delva R et al. (2010) Clinical value of circulating endothelial cells and circulating tumor cells in metastatic breast cancer patients treated first line with bevacizumab and chemotherapy. Ann Oncol 21: 1765-1771. doi:10.1093/annonc/mdq052. PubMed: 20233745. 20233745 10.1093/annonc/mdq052

[20] Fleitas T, Martínez-Sales V, Vila V, Reganon E, Mesado D et al. (2012) Circulating endothelial cells and microparticles as prognostic markers in advanced non-small cell lung cancer. PLOS ONE 7: e47365. doi:10.1371/journal.pone.0047365. PubMed: 23077602. 23077602 10.1371/journal.pone.0047365 PMC3471832

[21] Seaman S, Stevens J, Yang MY, Logsdon D, Graff-Cherry C et al. (2007) Genes that distinguish physiological and pathological angiogenesis. Cancer Cell 11: 539-554. doi:10.1016/j.ccr.2007.04.017. PubMed: 17560335. 17560335 10.1016/j.ccr.2007.04.017 PMC2039723

[22] Stupp R, Mason WP, van den Bent MJ, Weller M, Fisher B et al. (2005) Radiotherapy plus concomitant and adjuvant temozolomide for glioblastoma. N Engl J Med 352: 987-996. doi:10.1056/NEJMoa043330. PubMed: 15758009. 15758009 10.1056/NEJMoa043330

[23] Vredenburgh JJ, Desjardins A, Herndon JE 2nd, Dowell JM, Reardon DA et al. (2007) Phase II trial of bevacizumab and irinotecan in recurrent malignant glioma. Clin Cancer Res 13: 1253-1259. doi:10.1158/1078-0432.CCR-06-2309. PubMed: 17317837. 17317837 10.1158/1078-0432.CCR-06-2309

[24] Wen PY, Macdonald DR, Reardon DA, Cloughesy TF, Sorensen AG et al. (2010) Updated response assessment criteria for high-grade gliomas: Response assessment in neuro-oncology working group. J Clin Oncol 28: 1963-1972. doi:10.1200/JCO.2009.26.3541. PubMed: 20231676. 20231676 10.1200/JCO.2009.26.3541

[25] Chamberlain MC (2011) Radiographic patterns of relapse in glioblastoma. J Neuro Oncol 101: 319-323. doi:10.1007/s11060-010-0251-4. PubMed: 21052776. 10.1007/s11060-010-0251-4 21052776

[26] Norden AD, Young GS, Setayesh K, Muzikansky A, Klufas R et al. (2008) Bevacizumab for recurrent malignant gliomas: Efficacy, toxicity, and patterns of recurrence. Neurology 70: 779-787. doi:10.1212/01.wnl.0000304121.57857.38. PubMed: 18316689. 18316689 10.1212/01.wnl.0000304121.57857.38

[27] Mancuso P, Antoniotti P, Quarna J, Calleri A, Rabascio C et al. (2009) Validation of a standardized method for enumerating circulating endothelial cells and progenitors: Flow cytometry and molecular and ultrastructural analyses. Clin Cancer Res 15: 267-273. doi:10.1158/1078-0432.CCR-08-0432. PubMed: 19118054. 19118054 10.1158/1078-0432.CCR-08-0432

[28] Mead LE, Prater D, Yoder MC, Ingram DA (2008) Isolation and characterization of endothelial progenitor cells from human blood. Curr Protoc Stem Cell Biol Chapter 2: Unit 2C 1. PubMed: 18770637 10.1002/9780470151808.sc02c01s6 18770637

[29] Case J, Mead LE, Bessler WK, Prater D, White HA et al. (2007) Human CD34+AC133+VEGFR-2+ cells are not endothelial progenitor cells but distinct, primitive hematopoietic progenitors. Exp Hematol 35: 1109-1118. doi:10.1016/j.exphem.2007.04.002. PubMed: 17588480. 17588480 10.1016/j.exphem.2007.04.002

[30] Estes ML, Mund JA, Mead LE, Prater DN, Cai S et al. (2010) Application of polychromatic flow cytometry to identify novel subsets of circulating cells with angiogenic potential. Cytometry A 77: 831-839. PubMed: 20803735. 20803735 10.1002/cyto.a.20921 PMC2931367

[31] Mancuso P, Martin-Padura I, Calleri A, Marighetti P, Quarna J et al. (2011) Circulating perivascular progenitors: A target of PDGFR inhibition. Int J Cancer 129: 1344-1350. doi:10.1002/ijc.25816. PubMed: 21128230. 21128230 10.1002/ijc.25816

[32] Perry JR, Bélanger K, Mason WP, Fulton D, Kavan P et al. (2010) Phase II trial of continuous dose-intense temozolomide in recurrent malignant glioma: RESCUE study. J Clin Oncol 28: 2051-2057. doi:10.1200/JCO.2009.26.5520. PubMed: 20308655. 20308655 10.1200/JCO.2009.26.5520

[33] Beerepoot LV, Mehra N, Vermaat JS, Zonnenberg BA, Gebbink MF et al. (2004) Increased levels of viable circulating endothelial cells are an indicator of progressive disease in cancer patients. Ann Oncol 15: 139-145. doi:10.1093/annonc/mdh017. PubMed: 14679134. 14679134 10.1093/annonc/mdh017

[34] Lin M, Sutherland DR, Horsfall W, Totty N, Yeo E et al. (2002) Cell surface antigen CD109 is a novel member of the alpha(2) macroglobulin/C3, C4, C5 family of thioester-containing proteins. Blood 99: 1683-1691. doi:10.1182/blood.V99.5.1683. PubMed: 11861284. 11861284 10.1182/blood.v99.5.1683

[35] Hashimoto M, Ichihara M, Watanabe T, Kawai K, Koshikawa K et al. (2004) Expression of CD109 in human cancer. Oncogene 23: 3716-3720. doi:10.1038/sj.onc.1207418. PubMed: 15116102. 15116102 10.1038/sj.onc.1207418

[36] Greenberg JI, Shields DJ, Barillas SG, Acevedo LM, Murphy E et al. (2008) A role for VEGF as a negative regulator of pericyte function and vessel maturation. Nature 456: 809-813. doi:10.1038/nature07424. PubMed: 18997771. 18997771 10.1038/nature07424 PMC2605188

[37] Weisshardt P, Trarbach T, Dürig J, Paul A, Reis H et al. (2012) Tumor vessel stabilization and remodeling by anti-angiogenic therapy with bevacizumab. Histochem Cell Biol 137: 391-401. doi:10.1007/s00418-011-0898-8. PubMed: 22193946. 22193946 10.1007/s00418-011-0898-8

[38] Keunen O, Johansson M, Oudin A, Sanzey M, Rahim SA et al. (2011) Anti-VEGF treatment reduces blood supply and increases tumor cell invasion in glioblastoma. Proc Natl Acad Sci U S A 108: 3749-3754. doi:10.1073/pnas.1014480108. PubMed: 21321221. 21321221 10.1073/pnas.1014480108 PMC3048093

[39] Lu KV, Chang JP, Parachoniak CA, Pandika MM, Aghi MK et al. (2012) VEGF inhibits tumor cell invasion and mesenchymal transition through a MET/VEGFR2 complex. Cancer Cell 22: 21-35. doi:10.1016/j.ccr.2012.05.037. PubMed: 22789536. 22789536 10.1016/j.ccr.2012.05.037 PMC4068350

[40] Wong ET, Gautam S, Malchow C, Lun M, Pan E et al. (2011) Bevacizumab for recurrent glioblastoma multiforme: A meta-analysis. J Natl Compr Canc Netw 9: 403-407. PubMed: 21464145. 21464145 10.6004/jnccn.2011.0037

[41] Gil MJ, de Las Peñas R, Reynés G, Balañá C, Peréz-Segura P et al. (2012) Bevacizumab plus irinotecan in recurrent malignant glioma shows high overall survival in a multicenter retrospective pooled series of the spanish neuro-oncology research group (GEINO). Anti Cancer Drugs 23: 659-665. doi:10.1097/CAD.0b013e3283534d3e. PubMed: 22634799. 22634799 10.1097/CAD.0b013e3283534d3e

[42] Desjardins A, Reardon DA, Coan A, Marcello J, Herndon JE 2nd et al. (2012) Bevacizumab and daily temozolomide for recurrent glioblastoma. Cancer 118: 1302-1312. doi:10.1002/cncr.26381. PubMed: 21792866. 21792866 10.1002/cncr.26381

[43] Friedman HS, Prados MD, Wen PY, Mikkelsen T, Schiff D et al. (2009) Bevacizumab alone and in combination with irinotecan in recurrent glioblastoma. J Clin Oncol 27: 4733-4740. doi:10.1200/JCO.2008.19.8721. PubMed: 19720927. 19720927 10.1200/JCO.2008.19.8721

[44] Tchaicha JH, Reyes SB, Shin J, Hossain MG, Lang FF et al. (2011) Glioblastoma angiogenesis and tumor cell invasiveness are differentially regulated by beta8 integrin. Cancer Res 71: 6371-6381. doi:10.1158/0008-5472.CAN-11-0991. PubMed: 21859829. 21859829 10.1158/0008-5472.CAN-11-0991 PMC3193578

[45] Pope WB, Young JR, Ellingson BM (2011) Advances in MRI assessment of gliomas and response to anti-VEGF therapy. Curr Neurol Neurosci Rep 11: 336-344. doi:10.1007/s11910-011-0179-x. PubMed: 21234719. 21234719 10.1007/s11910-011-0179-x PMC3075404

[46] Gallego Perez-Larraya J, Lahutte M, Petrirena G, Reyes-Botero G, Gonzalez-Aguilar A et al. (2012) Response assessment in recurrent glioblastoma treated with irinotecan-bevacizumab: Comparative analysis of the macdonald, RECIST, RANO, and RECIST + F criteria. Neuro Oncol 14: 667-673. 22492961 10.1093/neuonc/nos070 PMC3337315

[47] Murray LJ, Bruno E, Uchida N, Hoffman R, Nayar R et al. (1999) CD109 is expressed on a subpopulation of CD34+ cells enriched in hematopoietic stem and progenitor cells. Exp Hematol 27: 1282-1294. doi:10.1016/S0301-472X(99)00071-5. PubMed: 10428505. 10428505 10.1016/s0301-472x(99)00071-5

[48] Bizet AA, Liu K, Tran-Khanh N, Saksena A, Vorstenbosch J et al. (2011) The TGF-beta co-receptor, CD109, promotes internalization and degradation of TGF-beta receptors. Biochim Biophys Acta 1813: 742-753. doi:10.1016/j.bbamcr.2011.01.028. PubMed: 21295082. 21295082 10.1016/j.bbamcr.2011.01.028

[49] Maharaj AS, Walshe TE, Saint-Geniez M, Venkatesha S, Maldonado AE et al. (2008) VEGF and TGF-beta are required for the maintenance of the choroid plexus and ependyma. J Exp Med 205: 491-501. doi:10.1084/jem.20072041. PubMed: 18268040. 18268040 10.1084/jem.20072041 PMC2271023

[50] Altman DG, McShane LM, Sauerbrei W, Taube SE (2012) Reporting recommendations for tumor marker prognostic studies (REMARK): Explanation and elaboration. PLOS Med 9: e1001216 PubMed: 2264269122675273. 22675273 10.1371/journal.pmed.1001216 PMC3362085

[51] Lambrechts D, Lenz HJ, de Haas S, Carmeliet P, Scherer SJ (2013) Markers of response for the antiangiogenic agent bevacizumab. J Clin Oncol 31: 1219-1230. doi:10.1200/JCO.2012.46.2762. PubMed: 23401453. 23401453 10.1200/JCO.2012.46.2762

