# Supplementary figures and images for: Prognostic Value of CD109+ Circulating Endothelial Cells in Recurrent Glioblastomas Treated with Bevacizumab and Irinotecan
Source: PLoS One. 2013 Sep 12;8(9):e74345. doi: 10.1371/journal.pone.0074345 (PMC3772091; doi:10.1371/journal.pone.0074345)

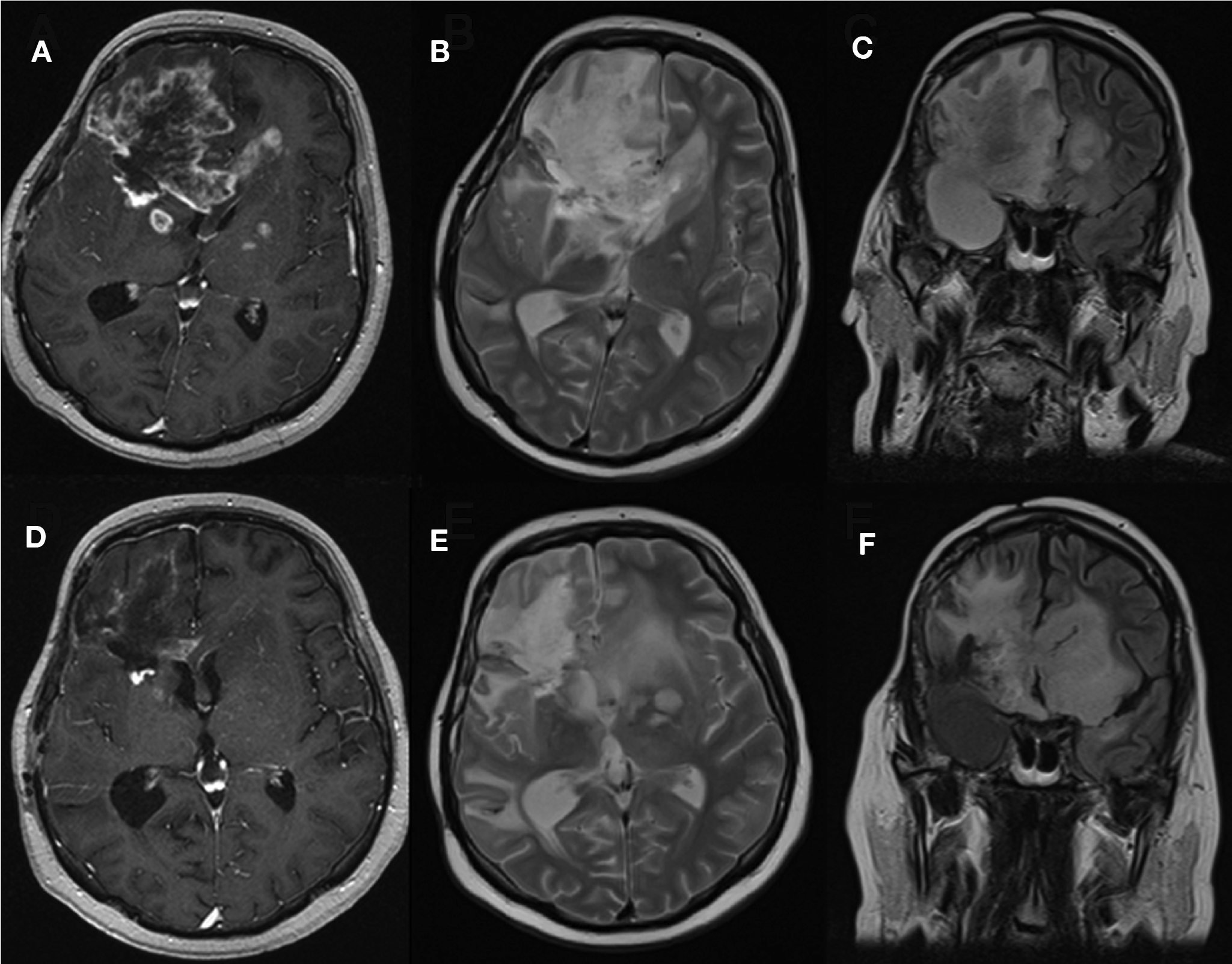

Supplement: Figure S1 — MRI of one patient with tumor progression (A, B, C before treatment. D, E, F at 2 months). From left to right: axial T1-weighted image (T1WI) with contrast injection, axial T2WI and coronal Flair image. A: recurrent GBM with irregular and marked enhancement and cystic-necrotic areas and invasion of genu of the corpus callosum. Small areas of enhancement are visible in the basal ganglia region bilaterally. B and C: the corresponding T2 and Flair showing heterogeneous hypersignal. The surgical cavity is visible in C. D: marked reduction of the enhancement in the left frontal region and corpus callosum, almost complete disappearance of enhancement in basal ganglia region and lowering of the mass effect. E and F: T2 hypersignal is increased and infiltration of contralateral frontal regions and a slight left hyperintensity in the temporal lobe are also visible. (TIF) [file pone.0074345.s001.tif]

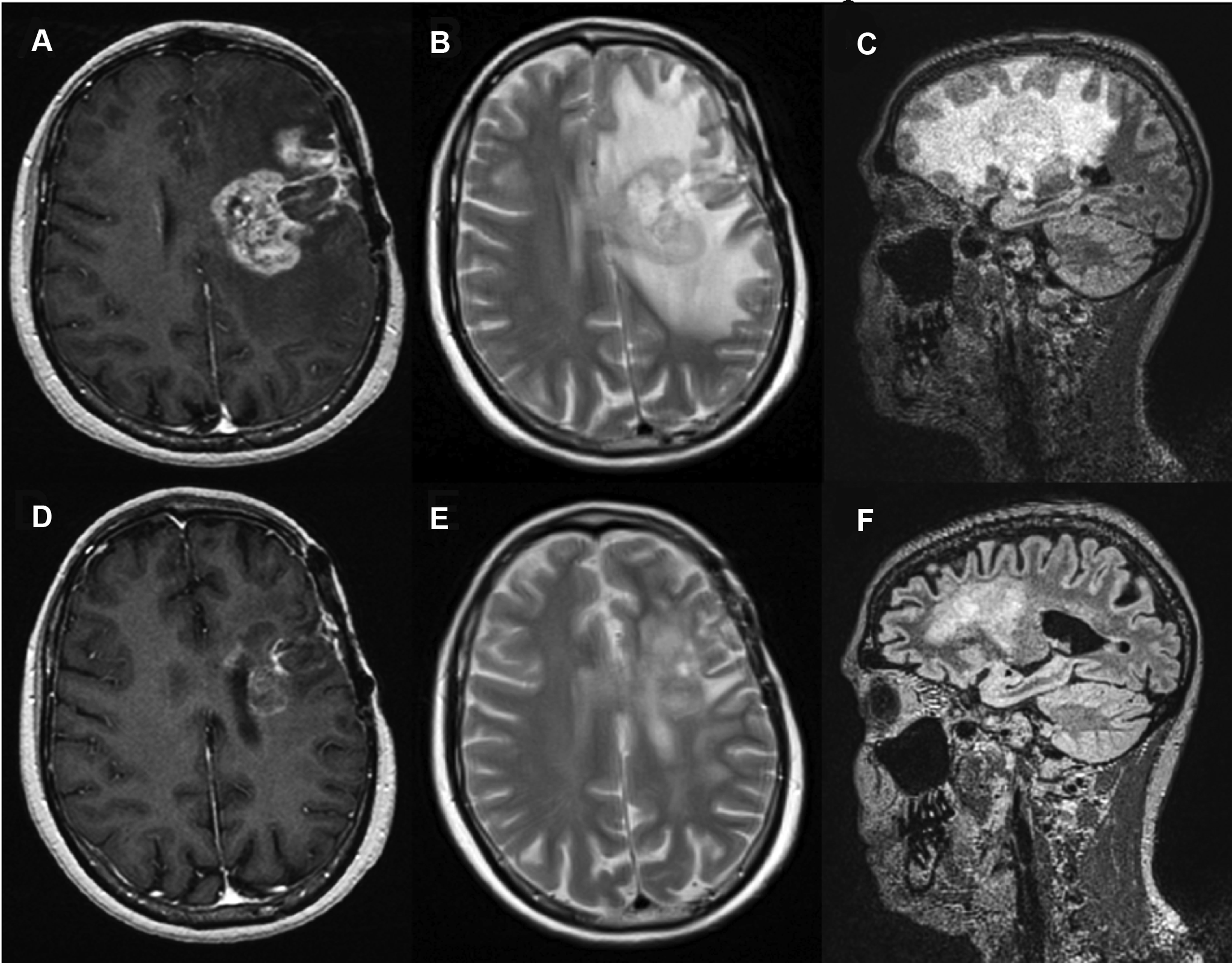

Supplement: Figure S2 — MRI of one patient responding to treatment (A, B, C before treatment. D, E, F at 2 months). From left to right: axial T1- weighted with contrast injection, axial T2-weighted, sagittal Flair. A: left frontal GBM characterized by strong and irregular enhancement. B: the tumor shows heterogeneous signal on T2-wi. C: On Flair images a large T2 hyperintensity surrounding the tumor is visible. The mass effect is demonstrated by narrowing of the cortical sulci and left lateral ventricle compression. D: after two months of therapy the enhancing tumor is dramatically reduced, the left lateral ventricle is slightly enlarged. E, F: only a small hyperintensity is seen on T2-wi and Flair images. No mass effect is visible and the sulci are clearly recognizable. (TIF) [file pone.0074345.s002.tif]

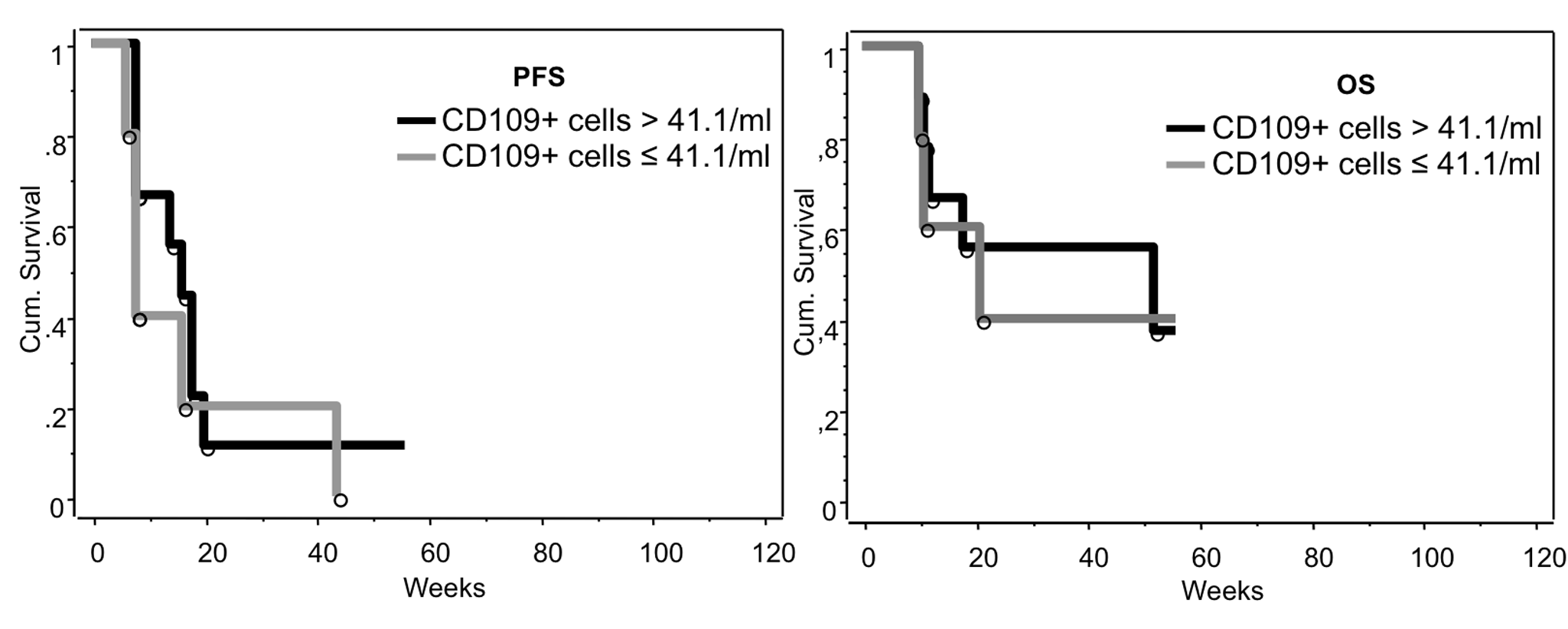

Supplement: Figure S3 — Baseline CD109+ CECs and PFS/OS in patients treated with classical antiblastic chemotherapy (IDB). Baseline CD109+ CEC count > 41.1/ml (1st quartile) were not associated with increased PFS or OS in IDB patients. (TIF) [file pone.0074345.s003.tif]
