# Supplementary material for: Prognostic Value of CD109+ Circulating Endothelial Cells in Recurrent Glioblastomas Treated with Bevacizumab and Irinotecan
Source: PLoS One. 2013 Sep 12;8(9):e74345. doi: 10.1371/journal.pone.0074345 (PMC3772091; doi:10.1371/journal.pone.0074345)
Supplement: Table S1 — IDA patient characteristics. (DOC) [file pone.0074345.s004.doc]

|  | | | |
| --- | --- | --- | --- |
| **Table S1A. IDA patient characteristics.** | | | |
| **Characteristic** | **No. of pts** |  | **%** |
| Gender | | | |
| Male | 21 |  | 66 |
| Female | 11 |  | 34 |
| Age, yrs | | | |
| Median [all pts] (range) |  | 53 (36-68) |  |
| < 40 | 2 |  | 6 |
| 40-60 | 22 |  | 69 |
| > 60 | 8 |  | 25 |
| KPS | | | |
| Median [all pts] (range) |  | 70 (50-90) |  |
| < 70 | 13 |  | 41 |
| 70-80 | 17 |  | 53 |
| 90-100 | 2 |  | 6 |
| Histological diagnosis | | | |
| De novo GBM | 30 |  | 94 |
| Secondary GBM | 2 |  | 6 |
| Time from 1^st^ diagnosis, mos (range) |  | 11 (7-92) |  |
| Disease recurrence | | | |
| 1^st^/2^nd^/3^rd^ | 32/7 |  | 100/22 |
| Prior therapy | | | |
| 1^st^/2^nd^/3^rd^ surgery | 32/8 |  | 100/25 |
| Radiotherapy | 32 |  | 100 |
| Radiosurgery | 1 |  | 3 |
| 1^st^/2^nd^/3^rd^ line chemotherapy | 32/7 |  | 100/22 |
| Systemic therapy | | | |
| No Dex/Dex<8mg/Dex≥8mg | 2/17/13 |  | 6/53/41 |
| EIAED therapy | 2 |  | 6 |
| Early progression according to RESCUE study ^18^ | - |  |  |
| PFS, wks | 18 (5-64) |  |  |
| PFS6 | 37 (95% CI 19-53) |  |  |
| OS, wks | 26 (5-64) |  |  |
| OS6 | 47 (95% CI 28-64) |  |  |
| OS12 | 25 (95% CI 7-42) |  |  |
| **Table S1B. IDB patient characteristics.** | | | |
| **Characteristic** | **No. of pts** |  | **%** |
| Gender | | | |
| Male | 6 |  | 43 |
| Female | 8 |  | 57 |
| Age, yrs | | | |
| Median [all pts] (range) |  | 54 (28-72) |  |
| < 40 | 1 |  | 8 |
| 40-60 | 10 |  | 71 |
| > 60 | 3 |  | 21 |
| KPS | | | |
| Median [all pts] (range) |  | 70 (50-90) |  |
| < 70 | 2 |  | 15 |
| 70-80 | 9 |  | 64 |
| 90-100 | 3 |  | 21 |
| Histological diagnosis | | | |
| De novo GBM | 13 |  | 92 |
| Secondary GBM | 1 |  | 8 |
| Time from 1^st^ diagnosis, mos (range) |  | 10 (6-61) |  |
| Disease recurrence | | | |
| 1^st^/2^nd^/3^rd^ | 14/4 |  | 100/29 |
| Prior therapy | | | |
| 1^st^/2^nd^/3^rd^ surgery | 14/4 |  | 100/29 |
| Radiotherapy | 14 |  | 100 |
| Radiosurgery | - |  | - |
| 1^st^/2^nd^/3^rd^ line chemotherapy | 14/4 |  | 100/29 |
| Systemic therapy | | | |
| No Dex/Dex<8mg/Dex≥8mg | 1/7/6 |  | 8/50/42 |
| EIAED therapy | - |  |  |
| Early progression according to RESCUE study ^18^ | - |  |  |
| PFS, wks | 14 (6-56) |  |  |
| PFS 6 | 7% (95% CI 6-20) |  |  |
| OS, wks | 21 (10-56) |  |  |
| OS6. | 49 (95% CI 23-76) |  |  |
| OS12 | 37 (95% CI 8-66) |  |  |
| Abbreviations: EIAED, enzyme-inducing anti-epileptic drugs; GBM, glioblastoma multiforme; mos, months; pts, patients; wks, weeks; yrs, years. | | | |
