# Supplementary material for: Prognostic Value of CD109+ Circulating Endothelial Cells in Recurrent Glioblastomas Treated with Bevacizumab and Irinotecan
Source: PLoS One. 2013 Sep 12;8(9):e74345. doi: 10.1371/journal.pone.0074345 (PMC3772091; doi:10.1371/journal.pone.0074345)
Supplement: Table S2 — Adverse events. (DOCX) [file pone.0074345.s005.docx]

| **Table S2. Adverse events.** | | |
| --- | --- | --- |
| **Adverse event** | **All grades** | **≥ grade 3** |
| Asthenia | 27 |  |
| Arterial hypertension | 23 |  |
| Diarrhea | 20 |  |
| Abdominal pain | 19 |  |
| Proteinuria | 17 |  |
| Vomiting | 14 | 1 |
| Nausea | 10 | 1 |
| Headache | 8 |  |
| Seizures | 5 | 1 |
| Leukopenia | 3 |  |
| Intralesional bleeding | 2 |  |
| Alopecia | 2 |  |
| Anemia | 2 |  |
| Deep venous thrombosis | 2 |  |
| Postsurgical sub-galeal infection | 2 | 2 |
| Anorexia | 1 | 1 |
| Cerebral sinus thrombosis | 1 | 1 |
| Conjunctiva bleeding | 1 |  |
| Epigastric discomfort | 1 | 1 |
| Epistaxis | 1 |  |
| Herpes infection | 1 | 1 |
| Hyperamilasemia | 1 | 1 |
| Ischemic heart failure | 1 | 1 |
| Pancreatic neoplasia | 1 | 1 |
| Sudden death | 1 | 1 |
| Suspected cerebral ischemic event | 1 | 1 |
| Vertebral fracture | 1 | 1 |
