# Supplementary material for: Prognostic Value of CD109+ Circulating Endothelial Cells in Recurrent Glioblastomas Treated with Bevacizumab and Irinotecan
Source: PLoS One. 2013 Sep 12;8(9):e74345. doi: 10.1371/journal.pone.0074345 (PMC3772091; doi:10.1371/journal.pone.0074345)
Supplement: Table S3 — Characteristics of patients with CD109+ CEC > 41.1/ml or ≤ 41.1/ml at baseline. (DOCX) [file pone.0074345.s006.docx]

| **Table S3. Characteristics of patients with CD109+CEC > 41.1/ml or ≤ 41.1/ml at baseline.** | | | |
| --- | --- | --- | --- |
|  | **CD109+CECs** | **CD109+CECs** |  |
| **Characteristics** | **> 41.1/ml** | **≤ 41.1/ml** |  |
| No. of pts | 48 | 17 |  |
| Age, yrs | | | |
| Median [all pts] (range) | 53 (15-68) | 51 (27-73) | n.s. |
| < 40 | 9 | 3 |  |
| 40-60 | 28 | 11 |  |
| > 60 | 11 | 3 |  |
| KPS | | | |
| Median [all pts] (range) | 70 (50-100) | 70 (50-100) | n.s. |
| < 70 | 13 | 5 |  |
| 70-80 | 32 | 11 |  |
| 90-100 | 3 | 1 |  |
| Histological diagnosis | | | |
| De novo GBM | 39 | 16 |  |
| Secondary GBM | 9 | 1 |  |
| Disease recurrence | | | |
| 1^st^/2^nd^/3^rd^ | 32/13/3 | 10/7 |  |
| Prior therapy | | | |
| 1^st^/2^nd^/3^rd^ surgery | 48/23 | 17/7 |  |
| Radiotherapy | 48 | 17 |  |
| Radiosurgery | 2 | 1 |  |
| 1^st^/2^nd^/3^rd^ line chemotherapy | 48/16/3 | 17/7/1 |  |
| Systemic therapy | | | |
| No Dex/Dex<8mg/Dex≥8mg | 6/19/23 | 2/4/11 |  |
| EIAED therapy | 6 | 2 |  |
| Tumor volume, cc (range) | 21.1 (0.97-127.1) | 36.7 (7.1-132.7) | n.s. |
| Early progression according to RESCUE study ^18^ | 4 | 2 |  |
| MRI patterns at baseline | | | |
| Local | 32 | 12 |  |
| Leptomeningeal dissem. | 8 | 2 |  |
| Distant | 8 | 2 |  |
| Multifocal | 0 | 1 |  |
| Abbreviations: cc, cubic centimetres; dissem., dissemination; EIAED, enzyme-inducing anti-epileptic drugs; GBM, glioblastoma multiforme; MR, magnetic resonance; pts, patients; yrs, years. | | | |
